# Cardiovascular risk markers (computed tomography‑coronary artery calcium and carotid intima‑media thickness) in patients with rheumatoid arthritis and controls

**DOI:** 10.3892/mi.2024.176

**Published:** 2024-07-12

**Authors:** Jalees Fatima, Vaibhav Shukla, Zeba Siddiqi, Mohammad Zakariya Shamsi, Saboor Mateen, Aaliya Abdul Jabbar, Zeenat Usmani

**Affiliations:** 1 Department of Medicine, Era's Lucknow Medical College and Hospital, Lucknow, Uttar Pradesh 226003, India; 2 Department of Cardiology, Mahatma Gandhi Medical College and Hospital, Jaipur, Rajasthan 302022, India

**Keywords:** rheumatoid arthritis, carotid intima-media thickness test, computed tomography-coronary artery calcium, early cardiac markers, rheumatology

## Abstract

Chronic inflammatory diseases, such as arthritis have been linked to a higher risk of developing cardiovascular disease. The present study examined the association between carotid intima-media thickness (CIMT) and coronary artery calcium (CAC), as well as the cardiovascular risk in patients with rheumatoid arthritis (RA). Additionally, the present study used 28 measures to calculate the disease activity score (DAS). To compare healthy controls with patients with RA, a case-control study was conducted that assessed CAC and CIMT in patients with the disease. A total of 45 healthy individuals and 45 patients with a diagnosis of RA were included in the study. With an average age of 50.66±12.35 years, the ages of the participants varied from 24 to 80 years. In both the control and RA patient groups, the sex ratio was 60%. The RA patient group had 53.3% female participants. There were significant variations in the levels of serum urea, potassium, magnesium, serum alkaline phosphatase, serum glutamic pyruvic transaminase, total leucocyte count, erythrocyte sedimentation rate, C-reactive protein (CRP) and lipids [apart from triglycerides and very low-density lipoprotein (VLDL)]. There was a substantial difference in the scores between patients with RA and the controls as regards CAC. A mild-severe risk of coronary artery disease was observed in 55.6% of RA cases and 4.4% of the controls (all mild). Both CIMT thickness and the CAC score exhibited a significant correlation with CRP, serum cholesterol, serum triglycerides, serum low-density lipids and serum VLDL. The DAS of patients ranged between 4.4 and 8.2 (mean, 5.81±0.91). A moderate disease activity was noted in the remaining patients, whereas 66.7% exhibited a high disease activity (DAS >5.2). On the whole, the present study demonstrates that conventional risk factors for cardiovascular disease, such as dyslipidemia, are consistent with both CIMT and CAC. The risk of developing atherosclerosis may be substantially increased by chronic inflammation, as the DAS score corresponds with CIMT and CAC.

## Introduction

Rheumatic illnesses are a general umbrella term for almost 200 different disorders affecting the musculoskeletal system (1). The causes and therapies for the almost 100 different types of arthritis have been well-documented. In humans, rheumatoid arthritis (RA) and osteoarthritis are the most well-known types of arthritis. RA, also referred to as an autoimmune disease or a progressive disease, is an example of a chronic systemic condition (2). It is characterized by destructive joint disease and prolonged inflammation, although its etiology is uncertain (3). There are two subtypes of this disease, namely ‘seropositive’ and ‘seronegative’. Elevated blood levels of rheumatoid factor (RF) autoantibodies and, more recently, antibodies to citrullinated protein/peptide antigens (ACPAs) can be used to determine seropositivity (4). RF autoantibodies are a type of antibody that are produced by the immune system.

RA is far more common in certain populations than in others. France (0.19%) and Italy (0.41%) have been found to have a lower reported prevalence of RA compared to other countries of the world, where it varies from 0.19 to 1.1% (5,6). The prevalence of RA in Germany and Sweden has been reported to be ~0.65% (7,8). RA has been reported to affect between 0.5 and 1% of North Americans (8-10). RA is estimated to afflict 0.75% of individuals in India (10,11).

Furthermore, coronary artery calcium (CAC) as it is commonly known, is a characteristic of coronary atherosclerosis (12). Consistent, dependable and convincing evidence of a substantial association between CAC and significant cardiovascular events has also been found in a number of asymptomatic individuals (13,14). Initially, CAC was evaluated using chest X-ray, fluoroscopy, or digital subtraction of fluoroscopy; following the introduction of electron beam computed tomography and multidetector computed tomography, these devices were found to be more useful. The most useful information on cardiovascular risk, or the likelihood of suffering a heart attack or stroke, may be obtained from coronary calcium scores for females aged 35 to 70 years and for males aged 40 to 60 years (15).

The disease activity score (DAS) was developed by a group of Dutch rheumatologists with the goal of offering a standardized method to compare and contrast results from clinical trials of novel medications for the treatment of RA. Clinical practices often use disease activity measurements related to RA. The DAS28, which evaluates a total of 28 joints, is one such instrument.

The present study aimed to examine the association between cardiovascular risk (using CAC) and CIMT in patients with RA and healthy controls, using the DAS28 as a proxy.

## Patients and methods

### Study population

The present case-control study was carried out at a tertiary care center over the course of 2 academic years (2020-2022). Each individual who met the American College of Rheumatology (ACR) criteria (Table SI) for a diagnosis of RA was included in the analysis as the cases, while those who were healthy, and age- and sex-matched individuals served as the controls. All patients were subjected to routine blood and radiological investigations. Patients with Inflammatory joint disease other than RA, a history of any cardiovascular/cerebrovascular events, known diabetes and obesity [body mass index (BMI) >30 kg/m^2^] and patients who were critically ill were excluded from the study. The Institutional Ethical Committee of Era's Lucknow Medical College and Hospital, Lucknow, India gave its approval for the research to go on. All patients were asked to provide their written and informed permission.

All controls and patients with RA who were identified using the aforementioned criteria were referred to the Radiology Department of the same hospital for evaluation of CIMT by carotid intima-media thickness ultrasonography and coronary artery calcium(CAC) burden by dual-energy computed tomography under the supervision of competent radiologists. These criteria categorize newly presented patients with confirmed clinical synovitis in at least one joint when no other medical condition seems to fit into the category. A score of ≥6 fulfils the requirements for definite RA. The classification criteria used in the present study for the patients with RA are listed in Table SI (16).

### DAS

Dutch rheumatologists first created the DAS in order to standardize and compare outcomes in RA medication clinical trials. Over time, the DAS28 has also found its way into everyday clinical settings. DAS28 is a metric for assessing the efficacy of RA therapy and management. Treatments may reduce the inflammation caused by RA, which in turn attenuates the damage inflicted to joints that causes disability and suffering. As a result, DAS28 (Table SII) (17) plays a crucial role in determining the optimal strategy for the disease management of each individual affected.

### Estimation of CAC score

The estimation of the CAC score was achieved using a 384-slice dual source machine, SOMATOM Force (Siemens Healthineers), for coronary calcium scoring at the Department of Radiology, Era's Lucknow Medical College and Hospital. The arteries which were focused on were the following: Left main, left circumflex, right circumflex and left anterior descending. The CAC score calculated using the Agatston score.

### Method of calculation

The calculation is based on the weighted density score given to the highest attenuation value (HU) multiplied by area of the calcification speck. The density factor assigned to each HU corresponds as 130-199 HU=1; 200-299 HU=2; 300-399 HU=3 and 400+ HU= 4. For example, if a calcified speck has maximum attenuation value of 400 HU and occupies 8 sq mm area, then its calcium score is 32. The severity is then graded as demonstrated in Table SIII (18).

### Statistical analysis

Statistical analysis was performed using SPSS version 21.0 statistical analysis software (IBM Corp.). The values are presented as number and percentage, or as the mean ± standard deviation (SD). Categorical variables were analyzed using the Chi-squared test or Fisher's exact test and continuous variables were analyzed using the unpaired student's t-test. Bivariate Pearson's (r-value) correlation analysis was used to determine the correlations among variables. A P-value <0.05 was considered to indicate a statistically significant difference.

## Results

**Figure 1:**
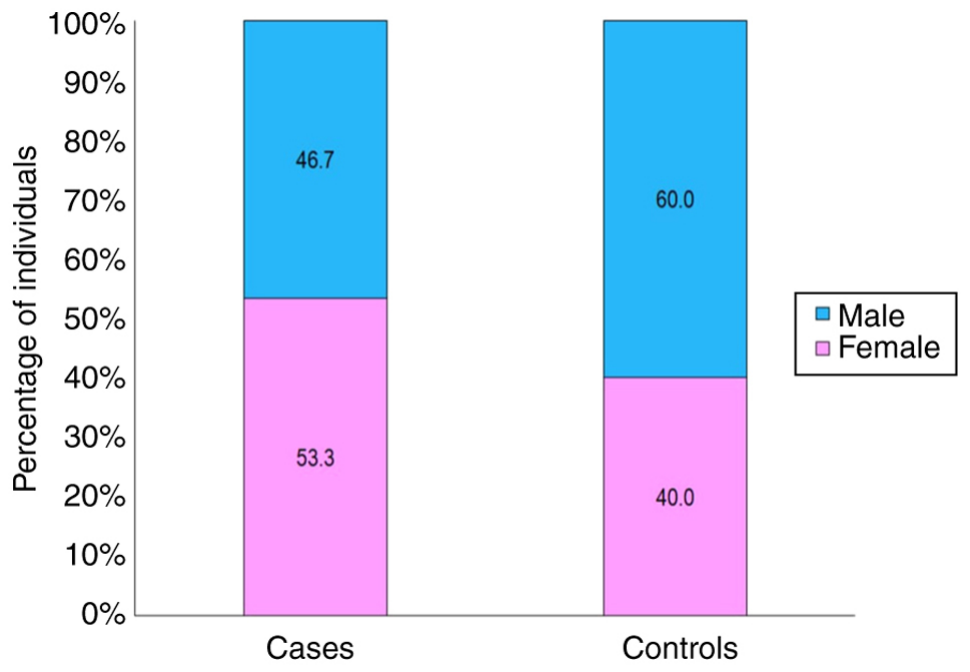
Comparison of the sex distribution between the cases and controls.

The present study examined 45 patients with RA who fulfilled the aforementioned inclusion criteria. These patients were then classified as the cases and another 45 healthy individuals were included as the controls. As demonstrated in Fig. 1, although the sex ratios between the patients with (53.3% females) and the controls (60% males) were comparable, the RA patient group had a higher number of females. The demographic profiles of both groups are presented in Table I.

Only 2 (4.4%) of the 45 patients with RA had a DAS ≤2.6, which is indicative of remission. A total of 40 patients of 45 cases (88.9%), had disease activity ratings that ranged from moderate to high (range, >3.2), whereas 3 patients, which is equivalent to 6.7% of the sample, had low disease activity scores (range, 2.6-3.2) (Table II).

Compared with the controls, the cases had a significantly higher total leucocyte count (TLC; 9311±2877 vs. 8049±1780 per microliter), erythrocyte sedimentation rate (ESR) in the first hour (19.18±6.34 vs. 16.62±3.56 mm/h), C-reactive protein (CRP; 11.36±5.84 vs. 2.44±1.69 mg/l), serum urea (24.76±7.96 vs. 21.99±3.53 mg/dl), serum potassium (4.18±0.69 vs. 3.95±0.23 mmol/l), serum magnesium (1.95±0.18 vs. 2.02±0.14 mg/dl), serum glutamic pyruvic transaminase (SGPT; 44.82±18.60 vs. 37.69±9.58 units/l), serum alkaline phosphatase (ALP; 96.02±36.12 vs. 81.96±21.19 IU/l), serum cholesterol (160.88±30.11 vs. 145.35±17.40 mg/dl), serum high-density lipoprotein (HDL; 7.20±15.83 vs. 43.91±8.17 mg/dl), serum low-density lipoprotein (LDL; 107.74±23.75 vs. 98.11±16.66 mg/dl) and the LDL-HDL ratio (2.85±1.58 vs. 1.57±3.27 mg/dl) (Table III). The results of the analysis of the correlation between the DAS, and BMI, CRP, serum cholesterol, serum triglycerides, serum HDL, serum LDL and serum very low-density lipoprotein (VLDL) are presented in Table IV. An inverse correlation was observed between the DAS and HDL levels. In addition, a strong correlation was found between the DAS and the CRP level. However, a moderate or weak correlation was observed between DAS and the remaining variables, although some correlations were significant.

None of the controls had moderate risk (score 101-400) or severe risk (score ≥401), and the majority of the controls exhibited no evidence of CAD (CAC score 0; 88.9%). The remaining controls had a minimum risk (score 1-10) or mild risk (score 11-100). By contrast, according to the CAC scores, only 22.2% of the patients with RA exhibited no signs of CAD, another 22.2% had a low risk, and the remaining 55.5% had a mild to severe risk. This difference was found to be statistically significant. The proportion of cases was higher as compared to controls in all the risk categories: Minimal (22.2 vs. 6.7%), mild (13.3 vs. 4.4%), moderate (24.4 vs. 0.0%) and severe risk (17.8 vs. 0.0%) (Table V).

The range of CIMT in the patients with RA was 0.712-1.310 mm, while that in the controls was 0.412-0.518 mm. In the patients with RA, the CIMT was determined to be 1.050±0.218 mm, which was substantially larger than the value of the control group (0.479±0.040 mm) (Table VI).

The CIMT exhibited a significant correlation with CRP, serum cholesterol, triglycerides, serum HDL (inverse), LDL and VLDL. A strong correlation was found between CIMT and CRP was strong; however, a moderate or weak correlation was found between CIMT and the remaining variables, although some correlations were significant. The CAC score also exhibited a significant correlation with CRP, serum cholesterol and serum triglycerides, serum VLDL and serum LDL. A moderate correlation was found between the CAC score and CRP, serum triglycerides and VLDL. The CAC scores and CIMT levels of both sexes were comparable. Age was not found to significantly correlate with the aforementioned two markers (CAC and CIMT) (Table VII).

## Discussion

As a chronic inflammatory joint condition, RA restricts everyday activities due to the pain, stiffness, and exhaustion that patients experience regularly. The link between inflammation and the development of cardiovascular disease was recognized only ~10 years ago. Researchers found that patients with RA had a 1.5-2.0-fold greater risk of developing coronary artery disease (CAD) than the general population (18). Another study demonstrated that patients with RA had a prevalence of myocardial infarction (MI) that was >3-fold higher than that of patients without RA (19). Thus, CAD is already more likely to occur before RA is even diagnosed clinically. The signs and symptoms of CAD vary with the disease process throughout time. In certain cases, there may be no obvious symptoms of damage.

The present study aimed to evaluate CIMT and computed tomography-CAC, two relatively novel cardiovascular risk indicators, in patients with RA. For this purpose, the present study recruited 45 patients with RA and 45 individuals who served as the healthy controls. The enrolled patients with RA had an average age of 49.07±12.38 years (ranging from 24 to 80 years), with 53.3% being female. Specifically, the controls in the present study were matched for both sex and age. Although RA may develop at any age, the majority of epidemiological research has shown that the condition is most common in individuals who are in their 50 and 60s. Other studies have suggested a later onset of the illness (19-23).

The mean age of patients with RA included in the clinical studies by Schott *et al* (24) (53.3 years) and Targońska-Stepniak *et al* (25) (42.6±8.0 years; range, 27-59) was also very close to that in the present study. In the study by Udachkina *et al* (26), the median age of the patients was 56 years.

In the present study, two-thirds (53.3%) of the patients had a DAS >5.2 (high disease activity), and16 patients had a DAS of 3.3-5.2 (35.6%) (moderate disease activity). Out of the 45 patients with RA, 2 (4.4%) were not on therapy, 6 (13.3%) were on disease-modifying antirheumatic drugs (DMARDs) treatment for <6 months, and the remaining 37 (82.22%) were on DMARDS treatment for >6 months. The DAS exhibited a substantial correlation with serum cholesterol, LDL and CRP levels among the CAD risk factors. Sengul *et al* (27) examined Turkish individuals with RA and their DAS28-ESR and DAS28-CRP criteria. While the DAS28-CRP was based on DAS28 without the ESR, the DAS28-ESR was based on the DAS with 28 joints (27). According to that study, the DAS28-CRP and DAS28-ESR exhibited a substantial association, whereas the individual components only exhibited a modest correlation (27).

In the present study, significant differences in the laboratory parameters (TLC, first-hour ESR, CRP, serum urea, serum potassium, serum magnesium, SGPT and serum ALP, and lipid levels) of the RA cases and healthy controls were observed. The TLC, ESR and CRP levels are inflammatory markers and have been found to be increased in ~40% of patients with RA (28). It has been shown that anti-inflammatory medications increase total, HDL and LDL cholesterol levels in patients with RA (29,30). Oxidative alterations caused by chronic inflammation modify the structure of HDL and decrease apolipoprotein-A1 in patients with active RA (31).

In the present study, the CAC score of the patients with RA was significantly higher compared with that of the controls (246.80±330.81 vs. 0.600±0.251). Of note, ~55.6% of patients with RA had been found to have a mild to severe risk of developing CAD (score, 11->400). A significant correlation between CAC and CRP, and lipid levels (apart from HDL) was found. These findings are supported by the findings in the study by Wahlin *et al* (32), who studied the computed tomography-CAC of 22 patients with RA (mean age, 65 years). They also found that 55.5% of the patients had a CAC score >10(32).

The average age of the 60 female patients with RA in the study by Bernardes *et al* (33) was 53.6±10.4 years, and their average DAS28 score was 4. Age, BMI, hyperglycemia and cholesterol levels were shown to have a substantial correlation with the mean coronary calcium score, which was 35.192±117.786. In the present study, the CIMT varied significantly (1.050±0.218 vs. 0.479±0.040 mm) between the experimental and control groups. CIMT was substantially linked with levels of lipids and CRP.

Targońska-Stepniak *et al* (25) found similar results when they evaluated CIMT values in 74 patients with RA without cardiovascular risk, with an average age of 46.4±0.6 years. A total of 31 age-matched control participants had notably higher CIMT values than the controls (25).

As a chronic inflammatory disorder, RA affects more than only the joints and muscles. Multiple risk variables that are also linked to cardiovascular risk govern it. Moreover, medication for RA also has a cardiovascular impact. Aging also contributes as a common risk factor between RA and cardiovascular disorders. Thus, the screening of all patients with RA for cardiovascular disease and associated risks is recommend at regular intervals. Furthermore, according to the data indicating a much larger percentage of these surrogate indicators in patients with RA, it is imperative that more stringent measures be implemented to decrease the cardiovascular risk in this population.

In conclusion, the present study demonstrated that the CIMT and CAC differed substantially between healthy individuals and patients with RA. This difference was shown to be statistically significant. The diagnosis and planning of risk reduction measures for patients with RA may be aided by these surrogate indicators of early atherosclerosis. Consistent results between CIMT and CAC, and established CV risk variables, such as dyslipidemia, suggest that these two methods could assist prevention and treatment efforts. Chronic inflammation significantly increases the risk of developing atherosclerosis, as suggested by the link between the disease activity score, the CIMT and CAC.

## Supplementary Material

Classification criteria for rheumatoid arthritis.

Disease activity score.

Severity grading of CAC score.

## Figures and Tables

**Table I:** Demographic details of the participants in the present study.

Demographic characteristics	Total (n=90)	Cases (n=45)	Controls (n=45)	t-test (P-value)
Mean age ± SD (range) in years	50.66±12.35	49.07±12.38	52.24±12.26	1.22 (0.224)
Mean BMI ± SD (range) in kg/m^2^	24.75±1.73	24.60±1.89	24.90±1.66	0.782 (0.436)

Cases, patients with rheumatoid arthritis; Controls, healthy controls; BMI, body mass index.

**Table II:** The comorbidities and therapy of the patients with RA.

Parameter	No. of cases	Percentage
Comorbidities		
Asthma	5	11.1
AKI	2	4.4
COPD	5	11.1
CKD	2	4.4
Hypothyroidism	7	15.6
Interstitial lung disease	4	8.9
No comorbidity	28	62.2
Therapy		
Not on therapy	2	4.4
On DMARDS treatment for <6 months	6	13.3
On DMARDS treatment for >6 months	37	82.2
DAS		
DAS <2.6 (remission)	2	4.4
DAS 2.6-3.2 (low disease activity)	3	6.7
DAS 3.3-5.2 (moderate disease activity)	16	35.6
DAS >5.2 (high disease activity)	24	53.3

RA, rheumatoid arthritis; AKI, acute kidney injury; COPD, chronic obstructive pulmonary disease; CKD, chronic kidney disease; DMARDS, disease-modifying antirheumatic drugs; DAS, disease activity score.

**Table III:** Comparison of the parameters between the cases and controls.

		Cases (n=45)		Controls (n=45)		Statistical significance	
Parameter	Average values	Mean	SD	Mean	SD	t value	P-value
Hemoglobin (mg/dl)	Males, 14-18 mg/dl; females,12-16 mg/dl	11.86	1.86	12.44	1.98	-1.438	0.154
TLC (cells/cumm)	4,000-11,000 cells/cumm	9311	2877	8049	1780	2.502	**0.014**
Platelets (lakh)	1.5-2.5 lakh	2.90	1.06	2.60	0.74	1.559	0.123
HbA1c (%)	Below 5.7%	5.42	0.40	5.30	0.53	1.215	0.227
RBS (mg/dl)	70-140 mg/dl	141.84	30.21	135.20	22.70	1.180	0.241
ESR in first-hour (mm/h)	0 to 15 mm/h in males; 0 to 20 mm/h in females	19.18	6.34	16.62	3.56	2.357	**0.021**
CRP (mg/dl)	<5 mg/l	11.36	5.84	2.44	1.69	9.840	**<0.001**
Serum urea (mg/dl)	5 to 20 mg/dl	24.76	7.96	21.99	3.53	2.138	**0.035**
Serum creatinine (mg/dl)	0.7 to 1.3 mg/dl	0.96	0.55	0.91	0.54	0.447	0.656
Serum sodium (mEq/l)	135 to 145 mEq/l	140.47	3.63	140.04	2.75	0.622	0.536
Serum potassium (mEq/l)	3.5 to 5.5 mEq/l	4.18	0.69	3.95	0.23	2.160	**0.033**
Serum magnesium (mg/dl)	1.6-2.5 mg/dl	1.95	0.18	2.02	0.14	-2.235	**0.028**
SGPT (U/l)	7 to 56 U/l	44.82	18.60	37.69	9.58	2.287	**0.025**
SGOT (U/l)	8 to 45 U/l	38.49	11.25	34.33	9.92	1.859	0.066
Serum total bilirubin (mg/dl)	0.1 to 1.2 mg/dl	0.93	0.30	0.91	0.30	0.248	0.805
Serum ALP (IU/l)	44 to 147 IU/l	96.02	36.12	81.96	21.19	2.253	**0.027**
Serum cholesterol (mg/dl)	<200 mg/dl	160.88	30.11	145.35	17.40	2.996	**0.004**
Serum triglycerides (mg/dl)	<150 mg/dl	179.69	34.94	176.64	16.34	0.530	0.598
Serum HDL (mg/dl)	>40 mg/dl	37.20	15.83	43.91	8.17	-2.527	**0.013**
Serum LDL (mg/dl)	<100 mg/dl	107.74	23.75	98.11	16.66	2.227	**0.029**
Serum VLDL (mg/dl) LD	2 to 30 mg/dl	35.94	6.99	35.33	3.27	0.530	0.598
LDL-HDL ratio	below 5:1	2.85	1.58	1.57	0.50	5.219	**<0.001**

Values in bold font indicate statistically significant differences (P<0.05). Cases, patients with rheumatoid arthritis; Controls, healthy controls; TLC, total leucocyte count; HbA1c, hemoglobin A1c; RBS, random blood sugar; ESR, erythrocyte sedimentation rate; CRP, C-reactive protein; SGPT, serum glutamic pyruvic transaminase; SGOT, serum glutamic-oxaloacetic transaminase; ALP, alkaline phosphatase; HDL, high-density lipoprotein; LDL, low-density lipoprotein; VLDL, very low-density lipoprotein.

**Table IV:** Correlation of DAS score with cardiovascular risk factors.

Cardiovascular risk factors	r value	Level of correlation	P-value
BMI	-0.055	Weak	0.606
CRP	0.730	Strong	**<0.001**
Serum cholesterol	0.319	Mild	**0.002**
Serum TGL	0.127	Weak	0.232
Serum HDL	-0.219	Weak	**0.038**
Serum LDL	0.462	Mild	**0.033**
Serum VLDL	0.127	Weak	0.232

Values in bold font indicate statistically significant differences (P<0.05). BMI, body mass index; CRP, C-reactive protein; TGL, triglycerides; HDL, high-density lipoprotein; LDL, low-density lipoprotein; VLDL, very low-density lipoprotein.

**Table V:** Comparison of CAC score between the groups.

		Cases (n=45)		Controls (n=45)		
CAC score	Total (n=90)	No. of participants	%	No. of participants	%	P-value (Fisher's exact test)
0 (No evidence of CAD)	50 (55.6)	10	22.2	40	88.9	**<0.001**
1-10 (Minimal)	13 (14.4)	10	22.2	3	6.7	**<0.001**
11-100 (Mild)	8 (8.9)	6	13.3	2	4.4	**<0.001**
101-400 (Moderate)	11 (12.2)	11	24.4	0	0.0	**<0.001**
≥401 (Severe)	8 (8.9)	8	17.8	0	0.0	**<0.001**
Mean CAC score ± SD (range)	123.70±263.49 (0-1,350)	246.80±330.81 (0-1,350)		0.600±0.251 (0-13)		N/A

Values in bold font indicate statistically significant differences (P<0.05). Cases, patients with rheumatoid arthritis; Controls, healthy controls; CAC, coronary artery calcium; CAD, coronary artery disease.

**Table VI:** Comparison of the CIMT between the groups.

Group	No. of subjects	Min.	Max.	Mean	S.D.	t-test (P-value)
Cases	45	0.712	1.310	1.050	0.218	17.277 (0.001)^a^
Controls	45	0.412	0.518	0.479	0.040	
Total	90	0.412	1.310	0.764	0.327	

^a^Indicates a statistically significant difference. Cases, patients with rheumatoid arthritis; Controls, healthy controls; CIMT, carotid intima-media thickness.

**Table VII:** Correlation of CIMT and CAC scores with cardiovascular risk factors.

	CIMT			CAC score		
Cardiovascular risk factors	r value	Level of correlation	P-value	r value	Level of correlation	P-value
BMI	-0.068	Weak	0.522	-0.046	Weak	0.664
CRP	0.880	Strong	**<0.001**	0.659	Moderate	**<0.001**
Serum cholesterol	0.319	Mild	**0.002**	0.306	Mild	**0.003**
Serum triglycerides	0.356	Mild	**0.001**	0.567	Moderate	<0.001
Serum HDL	-0.240	Weak	**0.023**	-0.45	Weak	0.671
Serum LDL	0.462	Mild	**<0.001**	0.336	Mild	**<0.001**
Serum VLDL	0.356	Mild	**0.001**	0.567	Moderate	**<0.001**
Age, years	0.145	Weak	0.174	0.058	Weak	0.590

Values in bold font indicate statistically significant differences (P<0.05). CAC, coronary artery calcium; CIMT, carotid intima-media thickness; BMI, body mass index, CRP, C-reactive protein; HDL, high-density lipoprotein; LDL, low-density lipoprotein; VLDL, very low-density lipoprotein.

## Data Availability

The datasets used and/or analyzed during the current study are available from the corresponding author on reasonable request.
