# Supplementary material for: Cardiovascular risk markers (computed tomography‑coronary artery calcium and carotid intima‑media thickness) in patients with rheumatoid arthritis and controls
Source: Med Int (Lond). 2024 Jul 12;4(5):52. doi: 10.3892/mi.2024.176 (PMC11273241; doi:10.3892/mi.2024.176)
Supplement: Classification criteria for rheumatoid arthritis. [file Supplementary_Data.pdf]

Table SI. Classification criteria for rheumatoid arthritis.

Criteria		Points
Joint involvement	1 Large joint (shoulder, elbow, hip, knee, ankle)	0
	2-10 Large joints	1
	1-3 Small joints (MCP, PIP, thumb, IP, MTP, wrists)	2
	4-10 Small joints	3
	>10 Joints (at least 1 small joint)	5
Serology	Negative RF and negative ACPA	0
	Low-positive RF or low-positive anti-CCP antibodies (≤3 times ULN)	2
	High-positive RF or high-positive anti-CCP antibodies (>3 times ULN)	2
Acute-phase reactants	Normal CRP and normal ESR	0
	Abnormal CRP or abnormal ESR	1
Duration of symptoms	<6 weeks	0
	≥6 weeks	1

MCP, metacarpophalangeal joint; PIP, proximal interphalangeal joint; IP, interphalangeal joint; MTP, metatarsophalangeal joint; RF, rheumatoid factor; ACPA, anti-citrullinated protein antibodies; CCP, cyclic citrullinated peptide; CRP, C-reactive protein; ESR, erythrocyte sedimentation rate.

Table SII. Disease activity score.

DAS28	Implication
<2.6	Disease remission. Usually no action necessary except remain on current medication.
2.6-3.2	Low disease activity. May merit change in therapy for some patients.
3.2-5.1	Moderate disease activity. May merit change in therapy for some patients unless mutually agreed to be the best outcome on current treatment.
>5.1	Severe disease activity likely to require change in therapy. This is the current threshold for being considered for biologic treatment as per the NICE guidelines.

Table SIII. Severity grading of CAC score.

0	(No evidence of CAD)
1-10	(Minimal)
11-100	(Mild)
101-400	(Moderate)
≥401	(Severe)

CAC, CAC, coronary artery calcium; CAD, coronary artery disease.
